# Au@Ag nanostructures for the sensitive detection of hydrogen peroxide

**DOI:** 10.1038/s41598-022-24344-w

**Published:** 2022-11-16

**Authors:** I-Hsiu Yeh, Sirimuvva Tadepalli, Keng-Ku Liu

**Affiliations:** 1 Department of Biomedical Engineering and Environmental Sciences, National Tsing Hua University, Hsinchu, 300044 Taiwan; 2 Microbiology and Immunology Department and Immunology Program, Stanford University School of Medicine, Stanford, CA 94305 USA

**Keywords:** Biochemistry, Biotechnology, Biomarkers, Diseases, Health care, Engineering, Materials science, Nanoscience and technology

## Abstract

Hydrogen peroxide (H_2_O_2_) is an important molecule in biological and environmental systems. In living systems, H_2_O_2_ plays essential functions in physical signaling pathways, cell growth, differentiation, and proliferation. Plasmonic nanostructures have attracted significant research attention in the fields of catalysis, imaging, and sensing applications because of their unique properties. Owing to the difference in the reduction potential, silver nanostructures have been proposed for the detection of H_2_O_2_. In this work, we demonstrate the Au@Ag nanocubes for the label- and enzyme-free detection of H_2_O_2_. Seed-mediated synthesis method was employed to realize the Au@Ag nanocubes with high uniformity. The Au@Ag nanocubes were demonstrated to exhibit the ability to monitor the H_2_O_2_ at concentration levels lower than 200 µM with r^2^ = 0.904 of the calibration curve and the limit of detection (LOD) of 1.11 µM. In the relatively narrow range of the H_2_O_2_ at concentration levels lower than 40 µM, the LOD was calculated to be 0.60 µM with r^2^ = 0.941 of the calibration curve of the H_2_O_2_ sensor. This facile fabrication strategy of the Au@Ag nanocubes would provide inspiring insights for the label- and enzyme-free detection of H_2_O_2._

## Introduction

Hydrogen peroxide (H_2_O_2_) is an important molecule in biological and environmental systems^1,2^. In living systems, H_2_O_2_ plays essential functions in physical signaling pathways, cell growth, differentiation, and proliferation^3^. H_2_O_2_ is considered as a neuromodulator in the central nervous system and immune system, and increasing evidences implicate that the H_2_O_2_ molecules can influence biological processes including signal transmission, immune response, embryonic development, and cell apoptosis^4^. Reactive oxygen species (ROS) such as H_2_O_2_ are widely regarded as a cytotoxic agent in cells. Numerous research works have revealed that the elevated level of H_2_O_2_ can cause severe damage in living cells. In the human body, the high level of H_2_O_2_ due to overproduction or lack of degradation is closely related to diseases including thyroiditis, tumorigenesis, and myxedematous cretinism^5^. Furthermore, H_2_O_2_ levels in blood have been reported to be linked to the Alzheimer’s disease and cancer^6,7^. In order to prevent harmful attacks to the cellular components, H_2_O_2_ levels must be precisely regulated by the antioxidant enzymes^8^. Therefore, a rapid and sensitive detection of H_2_O_2_ is vital in clinical diagnosis and bioanalysis.

Numerous strategies including spectrophotometry, electrochemistry, fluorescence, luminescence, and colorimetry methods have been proposed for detecting H_2_O_2_^1,9–16^. An enzyme-based sensing method is commonly employed as an optical technique for H_2_O_2_ detection^17,18^. Chromogenic substrates, such as 3,3′,5,5′-tetramethylbenzidine (TMB) and horseradish peroxidase (HRP), have been typically used for the measurement of H_2_O_2_ in colorimetric method. These sensing strategies provide unique merits including the high sensitivity and convenience. Recently, a great deal of research efforts have been dedicated to the nanozymes in order to improve the stability of the enzyme-based H_2_O_2_ sensors^19,20^. Among the nanomaterials with enzyme-like characteristics, noble metal nanostructures are promising nanomaterials for the enzyme-mimetic and enzyme-free detection of H_2_O_2_^21,22^. For example, graphene/Au-Pt nanostructures have been reported in recent research work for the sensitive detection of H_2_O_2_^23^. This nanozymes-based sensing method enables the in situ detection of H_2_O_2_ released from living cells^23^. Consequently, nanomaterials-based sensing methods with enzyme-mimetic activity make nanozymes promising techniques for the biomedical applications^21,24,25^. Recently, bimetallic nanoparticles have been employed for the sensing applications owing to their unique optical properties as well as their relatively simple preparation methods^26–28^.

**Figure 1:**
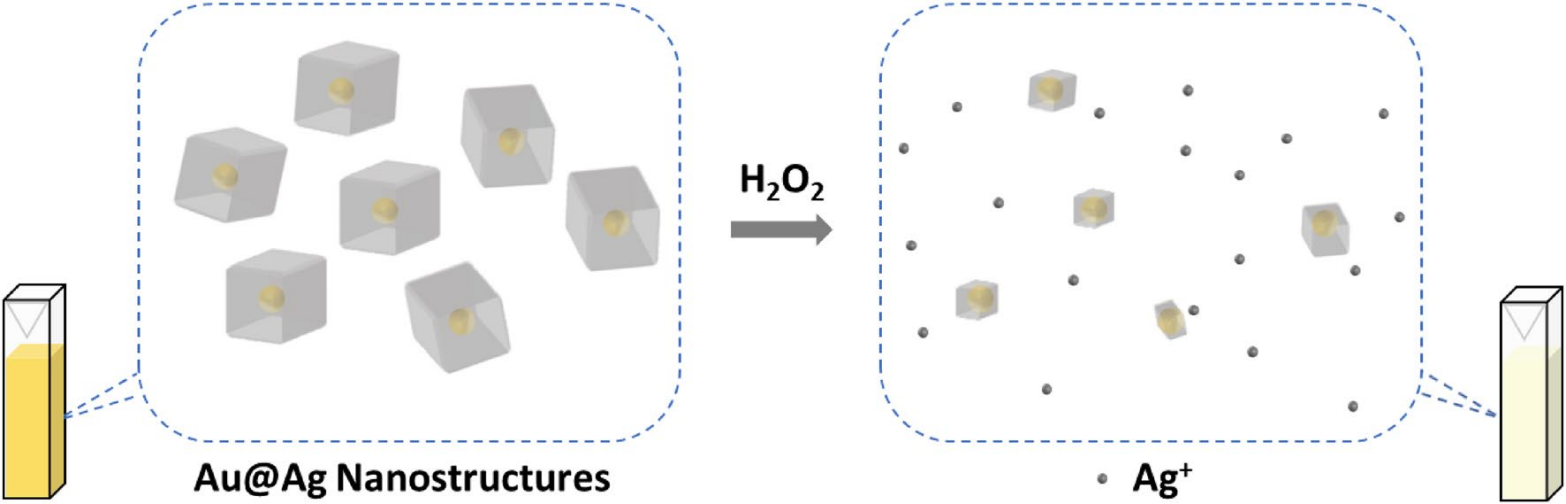
Schematic illustrating the concept of using Au@Ag nanocubes for the detection of H_2_O_2_.

In this work, we report the design and fabrication of the Au@Ag nanocubes for the label- and enzyme-free detection of H_2_O_2_ (Fig. 1). Seed-mediated synthesis method was employed to realize the Au@Ag nanocubes with high uniformity. The Au@Ag nanocubes were demonstrated to exhibit the ability to monitor the concentration of H_2_O_2_ at sub-micromolar concentration levels.

## Results

**Figure 2:**
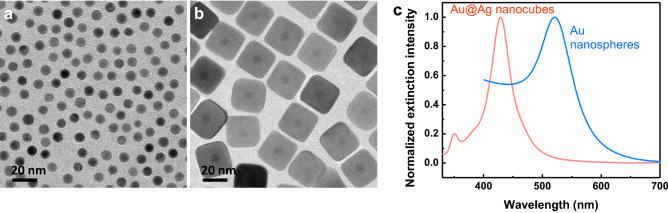
(**a**) TEM image of Au nanospheres. (**b**) TEM image of Au@Ag nanocubes. (**c**) Representative UV–Vis extinction spectra of the aqueous suspensions of Au nanospheres and Au@Ag nanocubes.

Owing to the relatively simple procedure and the aqueous solution process, a seed-mediated synthesis method that involved two-step process was employed in the synthesis of Au@Ag nanocubes. The synthesis of Au@Ag nanocubes starts with the synthesis of Au nanospheres, which serve as the core for the Ag coated Au nanospheres. Transmission electron microscopy (TEM) images reveal the size of the Au nanospheres with a diameter of 8.8 ± 0.4 nm (Fig. 2a). Au@Ag nanocubes are synthesized using Au nanospheres as the cores, this synthesis method is adapted from the recent reported procedures with slight modification (please see the experimental section for details)^29–31^. The growth solution is composed of silver nitrate as the silver precursor, ascorbic acid as the reducing agent, and the cetyltrimethylammonium chloride (CTAC) as the capping agent. TEM image reveals uniform size and shape of the Au@Ag nanocubes and the size was measured to be 31.8 ± 4.4 nm (as shown in Fig. 2b, Figures S1 and S2). The Au nanospheres were found to be at the center of the Au@Ag nanocubes, which indicating the uniform overgrowth of silver layer on the surface of the Au nanospheres. UV–Vis–NIR spectra reveal the localized surface plasmon resonance (LSPR) wavelength of the Au nanospheres is located at 521 nm, and the LSPR peak of Au@Ag nanocubes is located at 429 nm (Fig. 2c).

**Figure 3:**
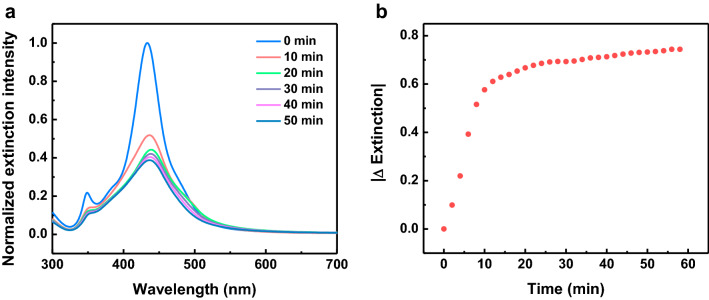
(**a**) Time-dependent UV–Vis extinction spectra of the aqueous suspensions of Au@Ag nanocubes in 200 µM H_2_O_2_. (**b**) Plot shows the changes of extinction of Au@Ag nanocubes as a function of the incubation time in H_2_O_2_.

We then turn our attention to the sensing performance of the Au@Ag nanocubes toward the H_2_O_2_. Owing to the difference of the reduction potential between the Ag^+^/Ag and the H_2_O_2_, Ag nanostructures were proposed to be able to serve as a reducing agent for the reduction of H_2_O_2_ (Table S1)^13^. On the basis of this oxidation–reduction reaction, the H_2_O_2_ induced degradation of Ag can lead to the decrease in the UV–Vis extinction intensity of the Au@Ag nanocubes. The Au@Ag nanocubes were incubated with H_2_O_2_ solution and the UV–Vis extinction spectra were collected at various time points. The time-dependent UV–Vis extinction spectra reveal that the extinction intensity is progressive decreasing with the incubation time (Fig. 3a). While small and no observable change in the |Δ Extinction| was observed when the Au nanomaterials were exposed to H_2_O_2_ with concentration of 200 µM (Figure S3). Plot shows the changes of the extinction intensity of Au@Ag nanocubes with the incubation time indicates the intensity of the peak change rapidly in the first 30 min and level off in 40 min (Fig. 3b). This observation suggested that the 40 min incubation time was sufficient for the reaction and we therefore selected 40 min incubation time in this assay.

**Figure 4:**
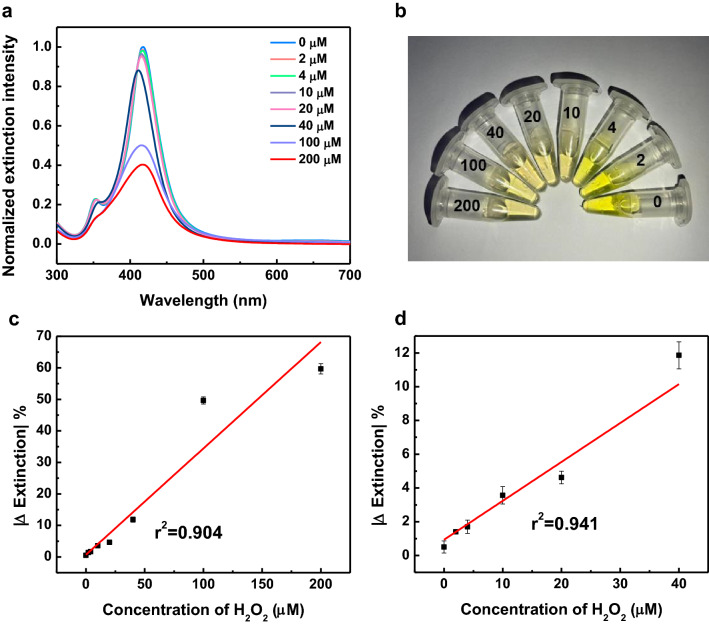
(**a**) UV–Vis extinction spectra of the aqueous suspensions of Au@Ag nanocubes in various concentrations of H_2_O_2_. (**b**) Optical image of the aqueous suspensions of Au@Ag nanocubes in various concentrations of H_2_O_2_ (unit: µM). (**c**) Plot shows the changes of extinction intensity of Au@Ag nanocubes as a function of the concentrations of H_2_O_2_ ranging from 0 to 200 µM and the linear fitting. (**d**) Plot shows the changes of extinction intensity of Au@Ag nanocubes as a function of the concentrations of H_2_O_2_ ranging from 0 to 40 µM and the linear fitting.

We further investigated the H_2_O_2_ concentration effect on the UV–Vis extinction spectra of the Au@Ag nanocubes. Various concentrations of H_2_O_2_ ranging from 0 to 200 µM were exposed to Au@Ag nanocubes, and the response of the H_2_O_2_ sensor showed that the intensity of the UV–Vis extinction spectra decreases gradually with the H_2_O_2_ concentration (Fig. 4a). Additionally, the full width at half maximum (FWHM) of the LSPR peak was getting broader as the UV–Vis extinction spectra decreases gradually with the H_2_O_2_ concentration. Colorimetric-based methods have been extensively used for rapid detection^32^. The color change of the Au@Ag nanocubes in the presence of various concentrations of H_2_O_2_ showed that the optical density decreases with the H_2_O_2_ concentration (Fig. 4b). Figure 4c shows the absolute value of change of the extinction intensity (|Δ Extinction|) of the Au@Ag nanocubes as a function of H_2_O_2_ concentration ranging from 0 to 200 µM. The calibration curve exhibits a linear relationship between the |Δ Extinction| and the H_2_O_2_ concentration (0 µM to 200 µM) with r^2^ = 0.904. The limit of detection (LOD, given by the average |Δ Extinction| at zero concentration (blank) plus three times of its standard deviation) was calculated to be 1.11 µM. In a relatively narrow range of the H_2_O_2_ concentration from 0 to 40 µM, the LOD was calculated to be 0.60 µM with r^2^ = 0.941 of the calibration curve of the H_2_O_2_ sensor (Fig. 4d). Recent reports revealed that the H_2_O_2_ concentration in blood plasma is about 1 µM to 5 µM, and high H_2_O_2_ concentration (≥ 10 µM) could induce cell death^20,33^. The LOD of our H_2_O_2_ sensing platform is lower than those concentrations, which suggests that the Au@Ag nanocubes can potentially serve as the nanozymes for rapid and sensitive detection of H_2_O_2_ in real world applications.

**Figure 5:**
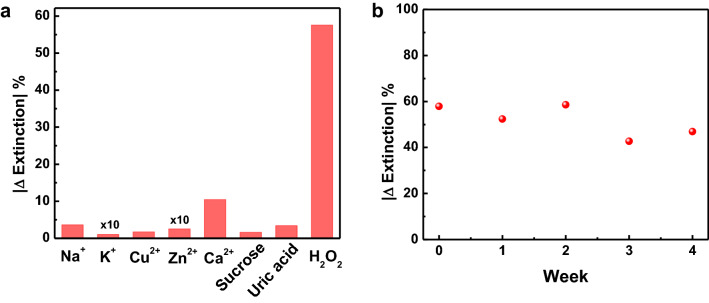
(**a**) Selectivity of the Au@Ag nanocubes sensor toward H_2_O_2_. (**b**) Stability of the Au@Ag nanocubes sensor toward H_2_O_2_ (200 µM).

To evaluate the selectivity of the H_2_O_2_ sensing platform, interference experiments were carried out using the species such as Na^+^, K^+^, Cu^2+^, Zn^2+^, Ca^2+^, sucrose and uric acid with the same concentration of H_2_O_2_ (200 µM). After exposure with these interfering species for 40 min incubation time, small and no observable change in the extinction spectra of Au@Ag nanocubes were found (Fig. 5a). While in the presence of the H_2_O_2_, an obvious extinction change was observed, confirming the high selectivity of this H_2_O_2_ sensing platform. We further investigated the stability of the Au@Ag nanocubes-based sensing platform toward the H_2_O_2_. For this test, we performed the H_2_O_2_ sensing experiments over four weeks and the |Δ Extinction| were recorded for each experiment. The |Δ Extinction| exhibited a remarkable stability over the time period tested, suggesting the excellent stability of this H_2_O_2_ sensing platform (Fig. 5b and Figure S4).

## Conclusions

In summary, we have demonstrated the Au@Ag nanocubes with well-controlled size and shape for the H_2_O_2_ detection. The Au@Ag nanocubes were demonstrated to exhibit the ability to monitor the H_2_O_2_ at concentrations lower than 200 µM with r^2^ = 0.904 and the limit of detection (LOD) of 1.11 µM. In the relatively narrow range of the H_2_O_2_ concentration from 0 to 40 µM, the LOD was calculated to be 0.60 µM with r^2^ = 0.941 of the calibration curve of the H_2_O_2_ sensor. In addition to the sensitivity at sub-micromolar concentration, the Au@Ag nanocubes exhibit excellent selectivity against numerous interfering species and long-term stability. This facile fabrication strategy of the Au@Ag nanocubes would provide inspiring insights for the label- and enzyme-free detection of H_2_O_2_. More broadly, the Au@Ag nanocubes are expected to be novel materials for the rapid and sensitive detection of H_2_O_2_ in clinical diagnosis and bioanalysis.

## Methods

### Materials

Cetyltrimethylammonium bromide (CTAB), gold chloride trihydrate (HAuCl_4_·3H_2_O), sodium borohydride, silver nitrate, and ascorbic acid were purchased from Sigma-Aldrich. Cetyltrimethylammonium chloride (CTAC) was purchased from Tokyo Chemical Industry (TCI). All the chemicals were used as received without further purification. Nanopure water (18.2 MΩ-cm) was used for all the experiments.

### Synthesis of Au nanospheres

Au nanospheres were synthesized using a previously reported procedure^34,35^. Au seeds were synthesized by adding 0.6 or 0.7 ml of ice-cold sodium borohydride solution (10 mM) into a solution containing 0.25 ml of HAuCl_4_ (10 mM) and 9.75 ml of CTAB (0.1 M) under vigorous stirring at room temperature. The color of seed solution changed from yellow to brown. Growth solution is composed of 6 ml of CTAC (0.2 M) ,4.5 ml of ascorbic acid (0.1 M), and 6 ml solution of HAuCl_4_ (0.5 mM). After stirring, 0.3 ml of the seed solution was added into the growth solution. The resulting solution containing Au nanospheres with diameter around 8 nm was centrifuged at 13,400 rpm for 30 min.

### Synthesis of Au@Ag nanocubes

Au@Ag nanocubes were synthesized using a previously reported procedure with slightly modification^29,30^. Briefly, 1 ml of Au nanospheres (extinction ~ 1.2) and 9 ml of CTAC (50 mM) were mixed at 60 °C under stirring for 20 min. 5 ml of AgNO_3_ (2 mM) and 2.5 ml of CTAC (80 mM) were added into the solution and stirring for 5 min. 2.5 ml of ascorbic acid (0.1 M) was added into the vial as a one-shot injection and the reaction was kept at 60 °C under stirring for 4 h. The Au@Ag nanocube solution was centrifuged and the nanostructures were redispersed in nanopure water.

### Detection of H_2_O_2_ with various concentrations

100 µl of freshly prepared H_2_O_2_ with various concentrations (from 1 mM to 10 µM) were added into 400 µl of twice-centrifuged Au@Ag nanocubes in nanopure water. The reaction solution was incubated for 40 min. After the incubation, UV–vis spectra were collected.

### Selectivity test

The selectivity of the Au@Ag nanocubes was tested with interferencing species such as Na^+^, K^+^, Cu^2+^, Zn^2+^, Ca^2+^, sucrose, and uric acid. 100 µl of interferencing species solution (1 mM) were added into 400 µl of twice-centrifuged Au@Ag nanocubes in nanopure water. The reaction solution was incubated for 40 min. After the incubation, UV–vis spectra were collected.

### Characterization techniques

Shimadzu UV-1900 spectrophotometer was employed for collecting UV–vis spectra. Scanning electron microscope (SEM) images were obtained using a JEOL JSM-7610F field emission instrument. Transmission electron microscope (TEM) images were obtained using a JEOL JEM-2100 field emission TEM. Atomic force microscopy (AFM) image was collected by Bruker Dimension Icon AFM in light tapping mode.

## Supplementary Information

Supplementary Information.

## Data Availability

The datasets used and/or analyzed during the current study available from the corresponding author on reasonable request.
