# Supplementary material for: Au@Ag nanostructures for the sensitive detection of hydrogen peroxide
Source: Sci Rep. 2022 Nov 16;12:19661. doi: 10.1038/s41598-022-24344-w (PMC9668984; doi:10.1038/s41598-022-24344-w)
Supplement: Supplementary file 1 — Supplementary Information. [file 41598_2022_24344_MOESM1_ESM.docx]

**Electronic Supplementary Information**

**Au@Ag nanostructures for the sensitive detection of hydrogen peroxide**

I-Hsiu Yeh ^†^, Sirimuvva Tadepalli ^‡^ and Keng-Ku Liu ^†^*

^†.^ Department of Biomedical Engineering and Environmental Sciences, National Tsing Hua University, Hsinchu 300044, Taiwan

^‡.^ Microbiology & Immunology Department and Immunology Program, Stanford University School of Medicine, Stanford, CA 94305, USA

*To whom correspondence should be addressed: kkliu@mx.nthu.edu.tw

**Table of Content**

Reactions of hydrogen peroxide and their standard redox potential ...…………………………. S-2

SEM image of Au@Ag nanostructures on SiO_2_/Si substrate ……………………………………. S-3

AFM image of Au@Ag nanostructures on SiO_2_/Si substrate ……………………………………. S-4

Normalized extinction spectra (before exposure to H_2_O_2_) of AuNP before and after exposure to H_2_O_2_ (200 µM) ...………………………………………………………………………………………. S-5

Normalized extinction spectra of Au@Ag nanostructures for the detection of H_2_O_2_ (200 µM) in the period of 4 weeks ...…………………………………………………………………………………... S-6

Table S1. Reactions of hydrogen peroxide and their standard redox potential.

pH condition Reaction of hydrogen peroxide Standard redox potential of H_2_O_2_

Acid H_2_O_2_ + 2 H^+^ + 2e^-^ $\to$ 2H_2_O E^0^=1.763 V

Base H_2_O_2_ + 2e^-^ $\to$ 2 OH^-^ E^0^=0.867 V

Source: *J. Phys. Chem. C*, 2010, 114, 6396-6400


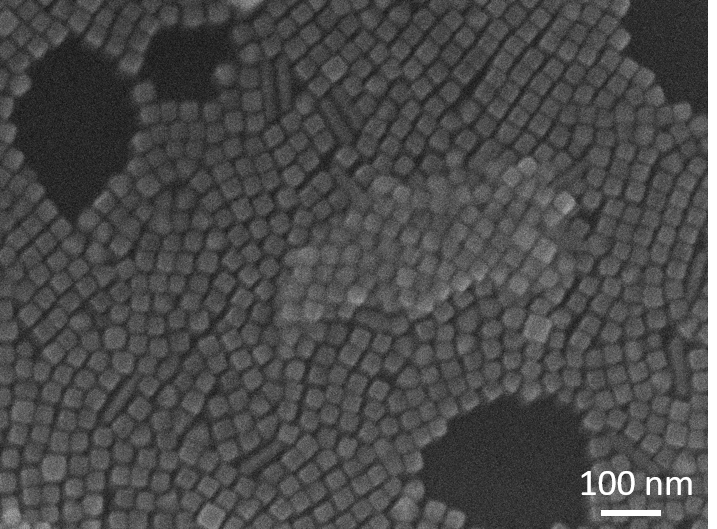


Fig. S1 SEM image of Au@Ag nanostructures on SiO_2_/Si substrate.


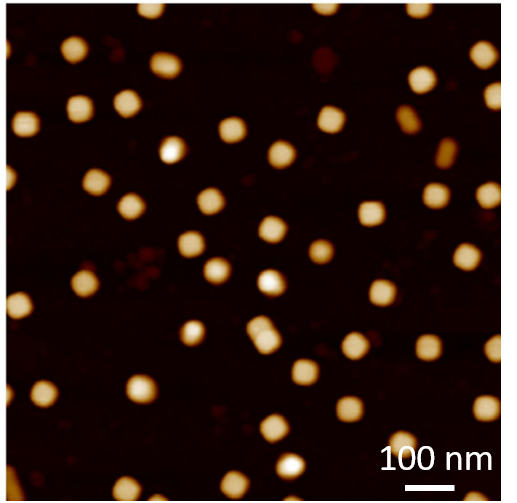


Fig. S2 AFM image of Au@Ag nanostructures on SiO_2_/Si substrate.

Fig. S3 Normalized extinction spectra (before exposure to H_2_O_2_) of AuNP before and after exposure to H_2_O_2_ (200 µM).


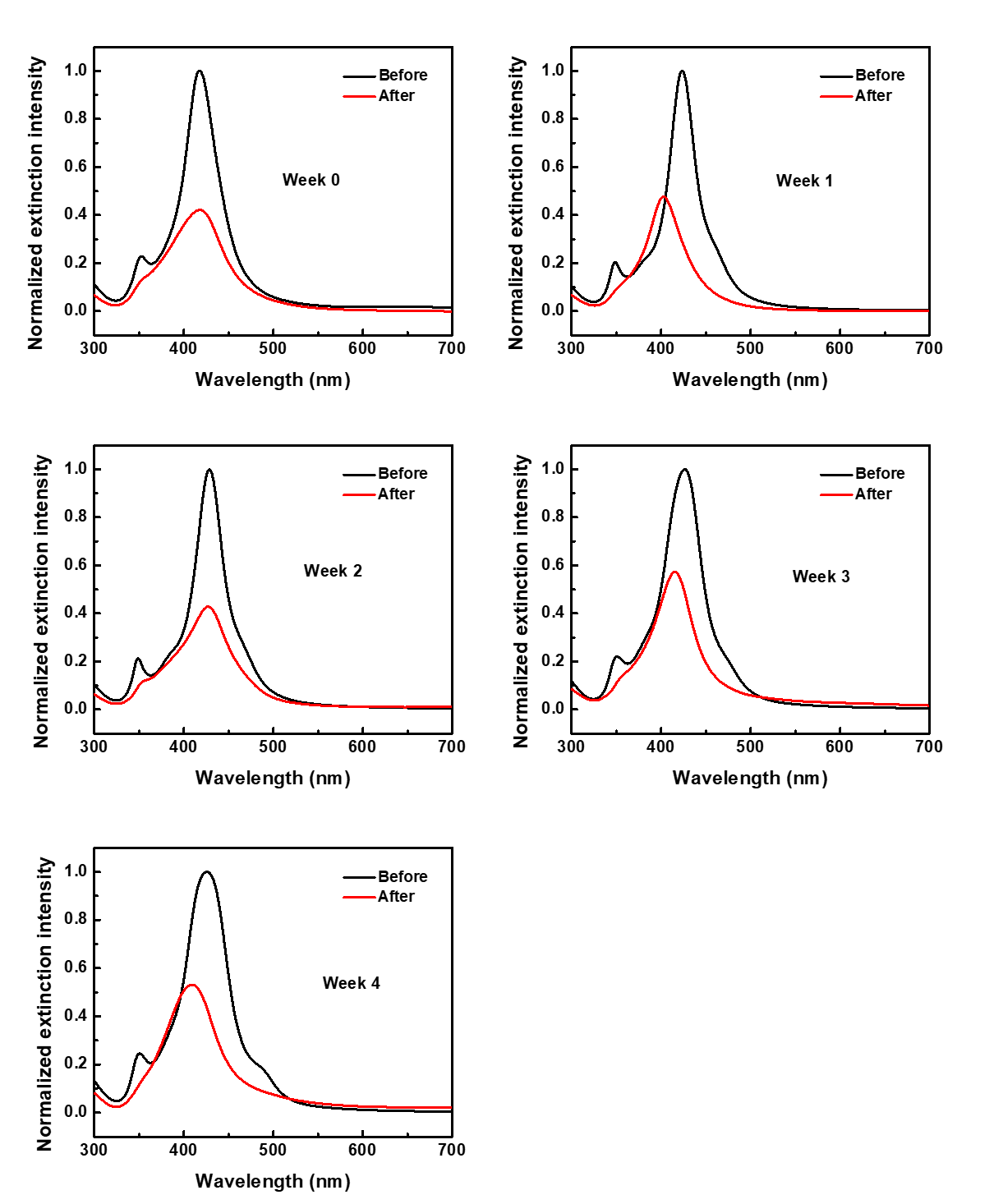


Fig. S4 Normalized extinction spectra (before exposure to H_2_O_2_) of Au@Ag nanostructures for the detection of H_2_O_2_ (200 µM) in the period of 4 weeks.
